# Analysis of Optic Nerve in Adults With Amblyopia Using OCTA

**DOI:** 10.3389/fmed.2022.903228

**Published:** 2022-07-14

**Authors:** Hui Lu, Tonggang Zhang, Tao Yue, Xiaoqin Li, Bingfen Ma, Hongxia Liu

**Affiliations:** ^1^Department of Ophthalmology, Zibo Central Hospital, Zibo, China; ^2^Department of Medical Device, Zibo Central Hospital, Zibo, China; ^3^Department of Gerontology, Zibo Central Hospital, Zibo, China

**Keywords:** adults, amblyopia, hyperopia, optical coherence tomography angiography, peripapillary microvasculature

## Abstract

**Objective:**

The aim was to quantify and compare papillary and peripapillary vessel density in amblyopic eyes of adults and age-matched controls.

**Methods:**

This cross-sectional study involved 20 eyes from 10 subjects with anisometropic amblyopia and 12 eyes of 6 age- and gender-matched healthy controls. Optical coherence tomography angiography (OCTA) was performed on all participants. SPSS 20 was used for data analysis.

**Results:**

The mean subject age was 35.7 ± 10.04 years (range 18–47) in the amblyopia group and 34.67 ± 6.92 years (range 23–42) in the control group. The diopter of amblyopia ranges from +3 to +5 ds. The mean inside optic disk capillary vessel density (CVD) was 41.88 ± 6.55% in amblyopic eyes, 49.23 ± 6.55% in fellow eyes, and 50.23 ± 4% in control eyes (*p* < 0.05). The mean inside optic disk all vessel density (AVD) was 52.97 ± 6.71% in amblyopic eyes, 59.87 ± 4.59% in fellow eyes, and 60.45 ± 2.8% in control eyes (*p* < 0.05). The amblyopic eye of participants showed a decrease in vessel density in the inside optic disk than in the fellow eyes and healthy subjects.

**Conclusion:**

Our present study revealed lower vessel density inside the optic disc of adult patients with anisometropic amblyopia. However, further studies are really needed to determine the clinical relevance of this finding.

## Background

Amblyopia is a common visual disorder, its character is subnormal visual acuity and contrast sensitivity in one or both eyes. Neurophysiological studies have demonstrated abnormal structural and functional alterations in the primary visual cortex and lateral geniculate nucleus in amblyopia (1). Also, although the controversial scientific evidence, retinal microstructural abnormalities have been reported in several studies using optical coherence tomography (OCT) (2–4). OCT angiography (OCTA) can provide vascular system visualization of the posterior segment in amblyopic eyes (5). At the same time, OCTA is an innovative and noninvasive technology, the patients can accept this examination easily.

The major purpose of our present study is to compare measurements of the radial peripapillary vascular plexuses among the amblyopic eyes of anisometropic amblyopic patients, their fellow eyes, and age- and sex-matched healthy control eyes.

## Materials and Methods

### Methods

This cross-sectional, comparative study was conducted at the department of ophthalmology of Zibo Central Hospital from January 2019 to December 2020. The study was approved by the Ethics Committee of Zibo Central Hospital and adhered to the tenets of the Declaration of Helsinki. Oral consent was obtained from the subjects after explanations of the study protocol.

### Study Subjects

A total of 20 eyes of 10 patients aged from 18 to 47 years with a diagnosis of amblyopia due to the hyperopia that was consecutively applied to the ophthalmology clinic were enrolled in this study. Amblyopia was defined as visual acuity of 20/30 or worse or a difference of two or more lines on Snellen's chart between better and worse eyes without organic cause for the decreased vision. The intraocular pressure (IOP) was normal in all subjects. Patients who had visual acuity of 20/20 uncorrected visual acuity (UCVA) and had no other ocular abnormality were enrolled in our study for the normal control group. We have 3 study groups: 10 amblyopic eyes of 10 amblyopic patients, 10 fellow eyes of 10 amblyopic patients, and 12 control eyes of 6 healthy controls. Exclusion criteria were as follows: media opacity, deprivation amblyopia, nystagmus, history of ocular disease, history of prematurity or intraocular surgery. Patients who have any systemic or neurological disorders were also not included.

### Study Examinations

The standard ophthalmologic examination had been performed on all participants; including slit lamp, fundus examinations, cycloplegic refraction (KR·800; TOPCON), and best-corrected visual acuity (BCVA). All participants underwent OCTA imaging by RTVue XR Avanti with AngioVue; Optovue Inc, Fremont, CA. OCTA has provided a detailed analysis of the optic disk's superficial and deep microvasculature. The optic disk OCTA images were obtained in 4.5 × 4.5 mm frames and contain 400 × 400 A-scans. The scans obtained were centered on the optic disk. We excluded images with significant motion artifacts.

### Statistical Analysis

For data analysis, SPSS 20 was used. Data presented in graphs are expressed as mean ± standard deviation (SD). Otherwise, the data is expressed as mean ± SD throughout. Data were analyzed using a *T*-test for two independent samples.

The significance of all analyses was considered as *p* ≦ 0.05.

## Results

The average age of 10 patients with anisometropic amblyopia was 35.7 ± 10.04 years, range 18–47 years, and five patients were men. We included 6 age- and sex-matched control subjects (34.67 ± 6.92 years, range 23–42 years). The ethnicity of all participants was Chinese.

### In 4.5 × 4.5 mm Optic Disk Scans

The amblyopic eyes were compared with the fellow eyes: the small vessels inside the optic disk in the amblyopic group were 41.88 ± 6.55%, and in the fellow group was 49.23 ± 6.55%, there was a statistically significant difference seen in the small vessels inside the optic disk between the amblyopic group and the fellow group (*P* < 0.05, Table 1). There was no statistically significant difference seen in the whole image and peripapillary small vessels between the amblyopic group and the fellow group (*P* > 0.05, Table 1). All vessels inside the optic disk in the amblyopic group were 52.97 ± 6.71%, and in the fellow group was 59.87 ± 4.59%, there was a statistically significant difference seen in all vessels inside the optic disk between the amblyopic group and the fellow group (*P* < 0.05, Table 2). There was no statistically significant difference seen in the whole image and peripapillary vessels between the amblyopic group and the fellow group (*P* > 0.05, Table 2). There was no statistically significant difference seen in the 8-sector peripapillary capillary small vessel density between amblyopic and fellow-subjects (*P* > 0.05, Table 3).

**Table 1:** Peripapillary capillary small vessel density in amblyopic and fellow subjects.

**Variables (%)**	**Amblyopic eyes (*n =* 10)**	**Fellow eyes (*n =* 10)**	***P*-value**
whole image	48.5 ± 2.32	49.3 ± 2.71	0.488
inside disk	41.88 ± 6.55	49.23 ± 6.55	0.022
peripapillary	50.86 ± 2.74	51.76 ± 3.27	0.513
superior-hemi	50.85 ± 3.10	52.55 ± 3.38	0.256
inferior-hemi	50.83 ± 2.87	50.90 ± 3.50	0.962

**Table 2:** Peripapillary capillary all vessel density in amblyopic and fellow subjects.

**Variables (%)**	**Amblyopic eyes (*n =* 10)**	**Fellow eyes (*n =* 10)**	***P*-value**
Whole image	55.04 ± 2.45	55.54 ± 2.57	0.661
Inside disk	52.97 ± 6.71	59.87 ± 4.59	0.015
Peripapillary	57.36 ± 2.49	57.61 ± 3.35	0.852
Superior-hemi	56.42 ± 3.69	58.21 ± 3.75	0.296
Inferior-hemi	57.26 ± 2.38	56.93 ± 3.44	0.806

**Table 3:** Eight-sector peripapillary capillary small vessel density in amblyopic and fellow-subjects.

**Variables (%)**	**Amblyopic eyes (*n =* 10)**	**Fellow eyes (*n =* 10)**	***P*-value**
Superior temporal	54.20 ± 3.85	56.80 ± 4.51	0.183
Superior	50.3 ± 5.21	51.00 ± 3.83	0.736
Superior nasal	47.00 ± 2.98	48.10 ± 4.82	0.547
Inferior nasal	49.80 ± 6.03	48.20 ± 5.55	0.545
Inferior	50.30 ± 3.86	50.30 ± 4.99	1.00
Inferior temporal	55.40 ± 4.95	52.90 ± 6.67	0.354
Temporal lower	48.80 ± 8.82	52.90 ± 6.01	0.240
Temporal upper	53.40 ± 10.05	56.20 ± 5.94	0.458

The amblyopic eyes were compared with the control eyes: the small vessels inside the optic disk in the amblyopic group were 41.88 ± 6.55% while it was 50.23 ± 4% in the control group, there was a statistically significant difference seen in the small vessels inside the disk between the amblyopic group and the control group (*P* < 0.01, Table 4). There was no statistically significant difference seen in the whole image and peripapillary small vessels between the amblyopic group and the control group (*P* > 0.05, Table 4). All vessels inside the optic disk in the amblyopic group were 52.97 ± 6.71%, and in the control group was 60.45 ± 2.8%, there was a statistically significant difference seen in all vessels inside the optic disk between the amblyopic group and the control group (*P* < 0.01, Table 5). There was no statistically significant difference seen in the whole image and peripapillary vessels between the amblyopic group and the control group (*P* > 0.05, Table 5). There was no statistically significant difference seen in the 8-sector peripapillary capillary small vessel density between the amblyopic and the control subjects (*P* > 0.05, Table 6).

**Table 4:** Peripapillary capillary small vessel density in amblyopic and control subjects.

**Variables (%)**	**Amblyopic eyes (*n =* 10)**	**Control eyes (*n =* 12)**	***P*-value**
Whole image	48.5 ± 2.32	50.31 ± 1.87	0.057
Inside disk	41.88 ± 6.55	50.23 ± 4.00	0.001
Peripapillary	50.86 ± 2.74	52.22 ± 2.26	0.218
Superior-hemi	50.85 ± 3.10	52.63 ± 2.27	0.135
Inferior-hemi	50.83 ± 2.87	51.78 ± 2.82	0.443

**Table 5:** Peripapillary capillary all vessel density in amblyopic and control subjects.

**Variables (%)**	**Amblyopic eyes (*n =* 10)**	**Control eyes (*n =* 12)**	***P*-value**
Whole image	55.04 ± 2.45	56.48 ± 1.75	0.125
Inside disk	52.97 ± 6.71	60.45 ± 2.80	0.002
Peripapillary	57.36 ± 2.49	58.17 ± 1.89	0.398
Superior-hemi	56.42 ± 3.69	58.78 ± 1.93	0.068
Inferior-hemi	57.26 ± 2.38	57.52 ± 2.25	0.798

**Table 6:** Eight-sector peripapillary capillary small vessel density in amblyopic and control subjects.

**Variables (%)**	**Amblyopic eyes (*n =* 10)**	**Control eyes (*n =* 12)**	***P*-value**
Superior temporal	54.20 ± 3.85	54.42 ± 4.14	0.901
Superior	50.3 ± 5.21	51.25 ± 4.79	0.661
Superior nasal	47.00 ± 2.98	48.75 ± 2.49	0.149
Inferior nasal	49.80 ± 6.03	47.25 ± 3.49	0.230
Inferior	50.30 ± 3.86	51.33 ± 3.80	0.535
Inferior temporal	55.40 ± 4.95	56.83 ± 3.16	0.420
Temporal lower	48.80 ± 8.82	53.25 ± 4.18	0.135
Temporal upper	53.40 ± 10.05	56.83 ± 3.69	0.284

**Figure 1:**
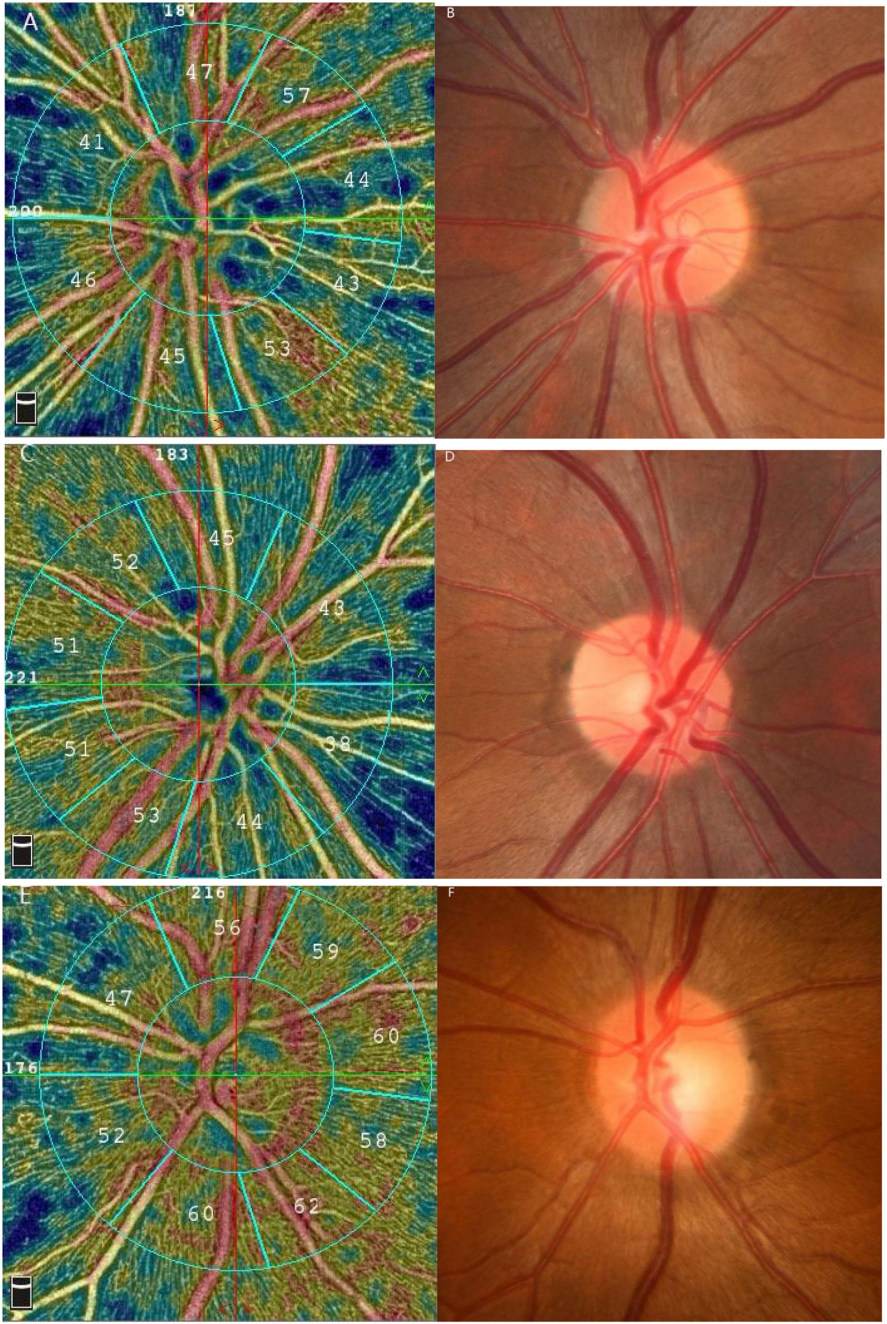
Results of the vessel density by 4.5 × 4.5 mm optic nerve scan and the fundus photographs. **(A,B)** the amblyopic eye **(C,D)** the fellow eye **(E,F)** the control eye.

The vessels inside the optic disk in the amblyopic eyes were lower than that of the fellow eyes and the control eyes, there is no obvious difference in fundus photography (Figure 1).

## Discussion

At present, the reliability and repeatability of OCTA technology have been confirmed by relevant studies (6–8). Di Antonio et al. (9) suggested that multiple *en face* OCTA image averaging is more reliable than a single image for the assessment of retinal functional parameters. The prevalence of amblyopia in China is ~1–3% (10). The results show that about 50% of amblyopia is caused by anisometropia, and about 40% by strabismus (11). In amblyopia, although the ocular examination is generally normal, a relationship between low vision and retinal microvasculature has been indicated previously (12), axial length also has an effect on peripapillary choroidal thickness in unilateral amblyopia (13).

A limited number of reports appear to have examined the CVD of the macular region and the optic disk using OCT-A in amblyopia (14, 15); however, only one of those studies include purely adult patients. The first study, including purely adult patients (20–65 years old), was conducted by Dereli Can (5), and they found significantly lower CVD in both the SCP and the DCP in amblyopic eyes of patients with amblyopia than in fellow eyes and healthy controls. Additionally, the area of FAZ measurements was significantly wider in amblyopic and fellow eyes than in control eyes. They advocate that decrease in microvascular density in both the SCP and DCP may be related to the reduced retinal ganglion cell size and number because the ganglion cells are located in the area of SCP feeding. At the same time, they concluded that the inside disk CVD was significantly lower in amblyopic patients; however, other OD OCTA parameters in the amblyopic group did not show significant differences compared with the control eyes.

Our study is the first one that evaluated the optic disk OCTA parameters in adult patients with amblyopia in China. Our current data illustrated a significantly lower vessel density inside the optic disc of patients with anisometropic amblyopia compared with the fellow eye and the control eye. To the present, our study is the first to analyze the blood flow density of all vessels in the optic disc of adult amblyopia patients. The blood flow density of all vessels in the optic disc may be related to the formation of amblyopia. At present, the drug treatment of amblyopia is mainly oral citicoline (16), there is no drug to improve microcirculation or vasodilator. In the treatment of amblyopia, the appropriate application of drugs to improve microcirculation and vasodilator may contribute to the improvement of visual acuity, which needs further research and exploration. In adulthood, treatment of amblyopia has long been widely known to be impossible; however, it has been recently shown that amblyopia can be treated with the aid of some special binocular perceptual learning programs, such as “Diploma Gabor Orientation Coherence Training” (17). For adult patients with amblyopia, it is worthy of clinical study whether giving appropriate drugs to dilate blood vessels and improve microcirculation will still improve vision.

Our study also showed that there was no significant difference in the blood flow density around the optic disk and in the 8-sector quadrant between the amblyopic eyes and the fellow eyes and the normal control group. This conclusion is consistent with that of Dereli Can (5). At the same time, Bayraktar et al. (18) had suggested that during routine clinical OCTA practice, the influence of moderate unilateral hyperopic anisometropic amblyopia on retinal vascular density measurements is relatively small and negligible. The formation of amblyopia may be related to the decrease of blood flow density in the optic disk.

There are only two studies on the changes in blood flow density in children with amblyopia before and after treatment. One study had the conclusion that amblyopic eye retinal vessel density (RVD) potentially increased after amblyopia treatment in specific regions of the retina (19). Another study indicated that anisometropic amblyopic eyes with lower macular vessel density could recover to the level of healthy eyes after full correction of the refractive errors and patching (20). The change of blood flow density in amblyopia before and after treatment is our next research goal.

## Conclusion

In summary, adult patients with amblyopia have lower vessel density inside the optic disc. These retinal vascular changes may be related to the pathogenesis of amblyopia. A prospective study with a larger sample size will be necessary to further explore this relationship and its clinical significance.

## Data Availability Statement

The original contributions presented in the study are included in the article/supplementary material, further inquiries can be directed to the corresponding author/s.

## Ethics Statement

The studies involving human participants were reviewed and approved by the Ethics Committee of Zibo Central Hospital. The patients/participants provided their written informed consent to participate in this study.

## Author Contributions

HLiu is the corresponding author, who contributed to the project design and the revision of the manuscript. HLu and TZ contributed to data analysis and interpretation and drafted the manuscript. TY contributed to specimen collection and manuscript discussion. XL and BM contributed to the OCTA examination of the subjects. All authors contributed to the article, read, and approved the submitted version.

## Funding

This work was supported by grants from the Key Research and Development Plan of Zibo City, No. 2019ZC010166.

## Conflict of Interest

The authors declare that the research was conducted in the absence of any commercial or financial relationships that could be construed as a potential conflict of interest.

## Publisher's Note

All claims expressed in this article are solely those of the authors and do not necessarily represent those of their affiliated organizations, or those of the publisher, the editors and the reviewers. Any product that may be evaluated in this article, or claim that may be made by its manufacturer, is not guaranteed or endorsed by the publisher.
